# Sterol Carrier Protein X (SCP-x) Regulates Cholesterol Transport in the Migratory Locust *Locusta migratoria*

**DOI:** 10.3390/biology15080613

**Published:** 2026-04-13

**Authors:** Dengbo Li, Tian Miao, Zige Wang, Zimeng Lang, Zixin Wang, Zixuan Zhou, Jinming Zhao, Panting Ma, Yuemin Ma

**Affiliations:** 1 College of Life Science, Shanxi University, Taiyuan 030006, China; 2 School of Synthetic Biology, Shanxi University, Taiyuan 030006, China

**Keywords:** *Locusta migratoria*, SCP-x, SCP-2, cholesterol

## Abstract

Sterol Carrier Protein X (SCP-x) is an evolutionarily conserved lipid transport protein that plays important roles in sterol metabolism. In insects, cholesterol is an essential component of cellular membranes and the precursor of ecdysteroids, yet insects cannot synthesize cholesterol de novo and must obtain it from dietary sources. However, the functional role of SCP-x in cholesterol absorption and transport in insects remains poorly understood. In this study, the SCP-x gene from the migratory locust *Locusta migratoria* was identified and characterized using transcriptomic data from the midgut and fat body. The full-length *LmSCP-x* encodes a 404-amino-acid protein containing both the 3-oxoacyl-CoA thiolase domain and the sterol carrier protein-2 domain. Expression analysis revealed that *LmSCP-x* is predominantly expressed in the midgut and fat body, and subcellular localization experiments showed that the protein is mainly distributed in the cytoplasm. RNA interference-mediated knockdown of *LmSCP-x* significantly reduced cholesterol levels in the fat body and delayed nymphal development. Structural prediction using AlphaFold 3 further revealed a conserved three-dimensional structure of the SCP-2 domain, and molecular docking identified key amino acid residues involved in cholesterol binding, which were subsequently validated by bio-layer interferometry assays. Together, these results demonstrate that *LmSCP-x* plays a crucial role in cholesterol transport in *L. migratoria* and provide new insights into sterol metabolism in insects, offering potential targets for the development of novel pest management strategies.

## Simple Summary

Cholesterol is essential for insects because it is required for building cell membranes and producing hormones that control growth and development. Unlike many other animals, insects cannot produce cholesterol on their own and must obtain it from their diet. However, how insects absorb and transport cholesterol within their bodies is still not fully understood. In this study, we investigated a gene in the migratory locust that is believed to be involved in cholesterol transport. We found that this gene is mainly active in the digestive organ and the fat storage tissue, two important sites for nutrient processing in insects. When the activity of this gene was reduced, the cholesterol level in these tissues decreased, and the insects developed more slowly. Structural analysis also suggested how the protein produced by this gene may interact with cholesterol molecules. These findings improve the understanding of how insects manage essential nutrients and may help scientists to develop more targeted and environmentally friendly methods to control harmful agricultural pests.

## 1. Introduction

Proteins, lipids, and carbohydrates are major energy sources supporting insect life activities. Among these, lipids play indispensable roles in key physiological processes such as growth and development, energy metabolism, environmental adaptation, reproduction, and population maintenance [1,2]. Lipids are widely distributed in various insect tissues, including the midgut, ovaries, and imaginal discs, whereas the fat body (FB) serves as the principal organ for lipid storage and metabolism. The fat body contains abundant lipid droplets and is responsible for lipid storage and mobilization [3]. Among different lipid types, cholesterol is particularly important for insect growth and development. It is a key component of cellular membranes, maintaining membrane fluidity, stability, and permeability, thereby ensuring proper material transport and signal transduction. In addition, cholesterol acts as a precursor for the synthesis of certain vitamins and hormones and participates in metabolic regulation and calcium–phosphorus homeostasis [4]. However, insects lack key enzymes required for cholesterol biosynthesis and therefore cannot synthesize cholesterol de novo. Instead, they must obtain sterols from their diet and rely on specialized transport proteins to mediate intracellular distribution and metabolism. Consequently, the uptake, transport, and metabolism of sterols are critical processes for insect growth and development.

Because cholesterol is highly hydrophobic, its intracellular transport within fat body cells cannot occur through free diffusion and instead requires specific carrier proteins [5]. Among these proteins, sterol carrier protein-x/2 (SCP-x/2) is a non-specific lipid transport protein widely distributed in eukaryotes, capable of binding a broad range of lipids with high affinity [6,7,8]. Notably, the SCP-x protein contains two characteristic domains: a 3-oxoacyl-CoA thiolase (SCP-t) domain and a sterol carrier protein 2 (SCP-2) domain, highlighting its multifunctional nature. The SCP-2 domain is primarily responsible for sterol binding and intracellular lipid transport, whereas the SCP-t domain mainly participates in fatty acid β-oxidation. SCP-x/2 was first identified in mammalian liver, and subsequent studies demonstrated that it also performs conserved and essential biological functions in insects. For example, SCP-2 is highly expressed in the midgut, fat body, and epidermis of several insects, including the *Helicoverpa armigera*, *Aedes aegypti*, and *Bombyx mori*, and its expression is closely associated with cholesterol absorption and transport [9,10,11]. SCP-2 interacts with cell membranes through positively charged surface regions, binds lipid molecules, and subsequently transfers them to target locations. This process depends on the conformational flexibility of the protein and significantly enhances the rate of sterol transport between membranes [12,13]. For instance, the spatiotemporal expression pattern of the *SlSCP-x* gene during larval development in the *Spodoptera litura* indicates a strong association with the midgut during feeding. Overexpression of this gene increases cholesterol uptake, whereas RNA interference-mediated suppression reduces cholesterol uptake and delays the transition from larva to pupa, demonstrating that this midgut-specific *SlSCP-x* gene plays a critical role in cholesterol uptake and normal development of the striped moth [14].

Numerous studies have investigated the protein structure of SCP-2 [15,16,17,18,19,20,21]. SCP-2 proteins typically have a molecular weight of approximately 13–16 kDa and possess a characteristic α/β-fold structure with a hydrophobic binding cavity. SCP-2 is a conserved protein domain widely present in many organisms and may exist either as an independent protein or as a functional domain within multifunctional proteins. Its core structure is highly stable and forms a dome-shaped fold composed of five β-strands and four α-helices. The turns between helices are often formed by glycine residues (Gly), which lack side chains and allow the protein backbone to rotate up to 180°, thereby facilitating conformational flexibility required for its biological function. SCP-2 contains a hydrophobic cavity that serves as the primary functional region responsible for binding and transporting hydrophobic lipid molecules such as fatty acids, cholesterol, and phospholipids. The size and shape of the SCP-2 cavity vary among different organisms, enabling adaptation to different ligands through conformational changes [15]. In some cases, SCP-2 can even bind multiple lipid molecules simultaneously. Through its highly conserved cholesterol-binding domain, SCP-2 can specifically recognize and bind cholesterol molecules within the hydrophobic pocket, thereby regulating the intracellular distribution of cholesterol. Specifically, SCP-2 mediates the uptake or reverse transport of cholesterol from cellular membranes or the endoplasmic reticulum to organelles such as mitochondria and the Golgi apparatus, participating in membrane assembly and hormone synthesis [22,23,24].

In recent years, due to the central role of SCP-2 in insect cholesterol metabolism and its relatively low toxicity in mammals, SCP-2 has emerged as an important target for the development of insect-specific inhibitors. Consequently, SCP-2 has attracted increasing attention in pest control research, particularly in the development of environmentally friendly pesticides. Ma H et al. resolved the three-dimensional structure of HaSCP-2 from the lepidopteran cotton bollworm using nuclear magnetic resonance (NMR) spectroscopy [18]. Sterol carrier protein inhibitors (SCPIs) competitively inhibit cholesterol binding to HaSCP-2 and significantly suppress cholesterol uptake in third-instar larvae, confirming that HaSCP-2 represents a promising insecticidal target for controlling resistant cotton bollworms [18]. Additionally, several curcumin derivatives have been reported to exhibit strong inhibitory activity against HaSCP-2 by occupying the sterol-binding pocket through hydrophobic interactions, thereby exerting insecticidal effects [25]. In *A. aegypti*, the sterol carrier protein 2-like protein AeSCP-2L2 has been structurally characterized using solution nuclear magnetic resonance, revealing key structural features involved in lipid binding. Furthermore, R. Barani Kumar et al. identified plant-derived larvicidal compounds targeting this protein through computational screening, providing a structural framework for the rational design of novel larvicides targeting mosquito SCP-2 proteins [19,26,27,28,29]. Although RNA interference (RNAi) has been widely used for functional gene studies, its application faces several limitations, including rapid degradation, poor cellular penetration, lack of targeting specificity, and rapid clearance, resulting in limited gene-silencing efficiency. Recently, nanocarrier-based delivery systems have emerged as promising strategies to improve RNAi efficiency. Nanocarriers can enhance gene-silencing effects by protecting RNA molecules, improving solubility, increasing targeting specificity, and facilitating endosomal escape. For example, fluorescent nanoparticles (FNPs) have been used as carriers for RNAi delivery to efficiently silence the HaSCP-2 gene in cotton bollworms, inhibiting cholesterol absorption and metabolism and significantly impairing growth, development, and reproduction, thereby providing a new approach for environmentally friendly pest control [30]. Kriska T et al. further demonstrated that SCP-2 not only transports physiological lipids but also mediates the transport of toxic lipid peroxides, expanding our understanding of SCP-2 function in oxidative stress and highlighting its potential value as a pharmacological target [31]. Similar to insects, many parasites also lack the de novo cholesterol biosynthesis pathway and must obtain cholesterol from their hosts via SCP-2-mediated transport. Because parasites are absolutely dependent on cholesterol, SCP-2 has also been proposed as a potential target for parasite control [32,33].

The migratory locust (*Locusta migratoria*) is a globally distributed agricultural pest characterized by extensive geographic distribution, high reproductive capacity, and severe crop damage, posing a persistent threat to food production and security. Currently, locust management mainly relies on chemical insecticides. However, the long-term and extensive use of these chemicals not only leads to environmental pollution and ecological imbalance but also accelerates the development of insecticide resistance in pest populations as well as the mortality of beneficial insects, including predators [34] and parasitoids [35]. Therefore, the development of efficient, environmentally friendly, and highly specific biological pesticides has become an urgent priority.

Despite these advances, studies on SCP-x in locusts remain limited. The specific role of SCP-x in sterol transport in locusts, its potential structural differences compared with other insects, and its feasibility as a target for locust pest control remain largely unexplored. In particular, systematic investigations into the molecular mechanisms underlying sterol transport mediated by SCP-x in *L. migratoria* (*LmSCP-x*) are still lacking. Therefore, this study aimed to investigate the function of the *LmSCP-x* gene and assess its potential application. A functional analysis system was established using molecular cloning, prokaryotic expression, antibody preparation, RNA interference, and molecular docking approaches. Furthermore, the bio-layer interferometry assay was employed to validate the binding of key amino acid residues to cholesterol, providing experimental evidence for the predicted protein–ligand interactions. The temporal and spatial expression patterns of *LmSCP-x* were characterized, and the molecular mechanism of *LmSCP-x* in sterol transport was analyzed. Future research may focus on developing more efficient nanocarrier systems to enhance targeted delivery in difficult-to-control pests such as locusts and promoting field applications of SCP-x-targeted strategies, thereby providing a theoretical basis and technical support for environmentally sustainable locust management.

## 2. Materials and Methods

### 2.1. Insect Rearing

Eggs of *L. migratoria* were obtained from a commercial breeding facility (*L. migratoria* Breeding Center, Cangzhou, Hebei Province, China), purchased via an online platform. Egg pods were placed in sandy soil in culture plates, sealed in transparent plastic bags with small pores for gas exchange, and incubated in a climate-controlled chamber (Shanxi University, Taiyuan, China) at 30 ± 1 °C and approximately 35% relative humidity. The soil was sprayed with water daily to maintain moisture.

After hatching, nymphs were transferred twice daily into metal cages (35 cm × 35 cm × 35 cm) and provided with fresh wheat seedlings ad libitum. The locusts were reared under a 16:8 h light:dark photoperiod at 30 ± 1 °C and 50–60% relative humidity. Uniform and healthy third-instar nymphs were selected for subsequent experiments.

### 2.2. Cell Line

The *Trichoplusia ni* cell line Tn-5B1-4 (Hi5) was obtained from Life Technologies (Carlsbad, CA, USA) and maintained in Grace’s insect medium (Life Technologies) supplemented with 10% fetal bovine serum, 100 U/mL penicillin, and 100 μg/mL streptomycin.

### 2.3. Phylogenetic Analysis of LmSCP-x

Sequence similarity analyses were conducted using BLASTP (NCBI BLAST+ v2.17.0). Theoretical molecular weight and isoelectric point were calculated using ProtParam (Expasy, https://web.expasy.org/protparam/, accessed on 9 April 2026). Multiple sequence alignment was performed using ClustalW v2.1. Phylogenetic analysis was conducted using MEGA 5.05. Amino acid sequences of LmSCP-x and homologous SCP-x proteins from other insects were aligned, and a phylogenetic tree was constructed using the neighbor-joining method with 1000 bootstrap replicates. Protein domain analysis was performed using the NCBI Conserved Domain Database, and the domain architecture was visualized using DOG software (Version 1.0) [36].

### 2.4. Plasmid Construction for Protein Expression in Hi5 Cells

Total RNA was extracted from the pooled midgut of five third-instar nymphs using RNAiso™ Plus (TaKaRa, Shiga, Japan) according to the manufacturer’s protocol. RNA integrity was verified by 1.5% agarose gel electrophoresis, and concentration was determined using a NanoDrop 2000 spectrophotometer (Thermo Fisher Scientific, Waltham, MA, USA). First-strand cDNA was synthesized from 2 μg total RNA using M-MLV reverse transcriptase (TaKaRa) and oligo(dT)18 primers.

The plasmid expressing LmSCP-x-GFP was constructed using the ClonExpress^®^ II One-Step Cloning Kit (Vazyme, Nanjing, China). Insert fragments and vector fragments (pie2-LmSCP--x-GFP) were amplified by PCR and purified using a Gel Extraction Kit (Bio-Tek, Beijing, China). Purified fragments were mixed and transformed into *E. coli* DH5α, where homologous recombination generated recombinant plasmids. Positive clones were verified by DNA sequencing. Primer sequences are listed in Supplementary Table S1. Plasmid DNA was purified using a Plasmid DNA Mini Kit (Omega Bio-Tek, Beijing, China).

### 2.5. Transfection and Microscopic Observation

Hi5 cells were seeded in six-well plates (Corning, Corning, NY, USA) at 2 × 10^6^ cells per well and cultured overnight. Cells were transfected with 2 μg plasmid DNA and 8 μL FuGENE HD reagent (Promega, Madison, WI, USA) according to the manufacturer’s instructions.

After 24 h, cells were fixed with 4% paraformaldehyde in 0.1 M phosphate-buffered saline (PBS) for 15 min. Nuclei were stained with Hoechst 33342 (1 μg/mL) for 10 min. Fluorescence images were obtained using a ZEISS LSM510 confocal microscope (ZEISS, Jena, Germany). GFP fluorescence and nuclear staining were merged to determine subcellular localization.

### 2.6. Expression and Purification of Recombinant Proteins

The LmSCP-2 fragment was cloned into a modified pRSFDuet-1 vector (Novagen, Madison, WI, USA) with an N-terminal His-SUMO tag using gene fusion strategies. Recombinant plasmids were verified by DNA sequencing and transformed into *Escherichia coli* Rosetta (DE3) cells. Cells were cultured at 37 °C until OD600 ≈ 0.6, followed by induction with 0.5 mM IPTG at 18 °C for 16 h. Cells were harvested by centrifugation (5000× *g*, 6 min), resuspended in lysis buffer (20 mM Tris-HCl, 200 mM NaCl, pH 8.0), and disrupted using a cell disruptor (JNBIO, Guangzhou, China).

After centrifugation, the supernatant was applied to a Ni^2+^-Sepharose column (HisTrap HP, GE Healthcare, Chicago, IL, USA). The column was washed with 20 mM imidazole buffer, and proteins were eluted with 250 mM imidazole buffer. The His-SUMO tag was removed by Ulp1 protease digestion at 4 °C overnight. Further purification was performed using Superdex 200 gel filtration (Cytiva, Wilmington, DE, USA). Protein purity was assessed by SDS–PAGE. The primers used for expression of LmSCP-2 fragment are listed in Table S2.

### 2.7. Immunofluorescence Detection of LmSCP-x in Tissue Sections

Immunofluorescence detection of LmSCPx in tissue sections was performed following Mollah et al. [37] with some modifications. In brief, midgut tissues from third-instar nymphs were dissected and fixed in 4% paraformaldehyde, followed by paraffin embedding and sectioning using a microtome (Leica, Nussloch, Germany). Sections were mounted on salinized slides, dewaxed, and rehydrated. Antigen retrieval was performed using 10 mM citrate buffer (pH 6.0) at 98 °C for 10 min. Sections were blocked with 5% goat serum and incubated with anti-LmSCP-x primary antibody (4.4 μg/mL) and then with goat anti-rabbit secondary antibody (2.5 μg/mL). Nuclei were stained with SYTOX™ Green (1:5000) for 15 min in the dark. Fluorescence signals were visualized using a fluorescence microscope (Leica Microsystems, Wetzlar, Germany). The polyclonal LmSCP-x antibody was generated by ABclonal Biotechnology Co., Ltd. (Wuhan, China) using purified LmSCP-2 protein. Three midguts per group were analyzed, and three images were captured from each tissue.

### 2.8. Tissue Expression Analysis by RT-qPCR

Tissues were collected from ten independent third-instar nymphs. Each sample included three biological replicates. Tissues were frozen in liquid nitrogen and stored at −80 °C before RNA extraction and cDNA synthesis.

RT-qPCR was performed using a Bio-Rad Real-Time PCR system with SYBR Green Master Mix (TOYOBO, Tokyo, Japan) following Mollah and Kim [38] with some modifications. Each reaction (15 μL) contained 4 μL diluted cDNA, 0.6 μL of each primer (10 μM), 7.5 μL SYBR mix, and 3.3 μL nuclease-free water. PCR conditions were: 95 °C for 15 s, 40 cycles of 95 °C for 15 s and 60 °C for 31 s. Specificity was confirmed by melt curve analysis. EF1α was used as the internal reference gene. Relative expression levels were calculated using the 2^−ΔCt^ method. Primer sequences are listed in Table S4.

### 2.9. RNA Interference and Bioassay

#### 2.9.1. dsRNA Preparation

Double-stranded RNA (dsRNA) was synthesized following the method described by Mollah et al. [39] using primers containing the T7 promoter sequence (Table S3). dsRNA targeting *LmSCP-x* and *GFP* (control) was generated using the T7 RiboMAX™ Express RNAi System (Promega, Madison, WI, USA). dsRNA concentration was adjusted to 2 μg/μL, and integrity was verified by 1% agarose gel electrophoresis.

#### 2.9.2. RNAi Treatment

A total of 3 μL dsLmSCP-x solution (6 μg dsRNA) was injected into the abdominal intersegmental membrane of one-day-old third-instar nymphs using a microinjector. Control insects received dsGFP. Each group contained three biological replicates, with eight insects (4♀ + 4♂) per replicate. At 48 h post-injection, integuments were dissected for RNA extraction and RT-qPCR analysis to determine gene knockdown efficiency.

#### 2.9.3. Bioassay and Cholesterol Measurement

To evaluate the role of LmSCP-x in cholesterol transport, 10 third-instar nymphs (1:1 female:male) were injected with 6 μg dsLmSCP-x, while controls received dsGFP. After 24 h, knockdown efficiency was assessed and the cholesterol levels in midgut and fat body tissues were measured.

Cholesterol concentration was determined using a Total Cholesterol Assay Kit (Applygen, Beijing, China). Total protein content was measured using a BCA Protein Assay Kit (Thermo Scientific, Waltham, MA, USA). Relative cholesterol levels were calculated as: cholesterol concentration (mg/mL)/total protein (mg). Three biological replicates were performed.

For Oil Red O staining, fat body tissues were embedded in OCT compound and sectioned at 10 μm thickness at −20 °C. Sections were stained using a Modified Oil Red O Stain Kit (Solarbio, Beijing, China) and counterstained with hematoxylin–eosin (HE). Images were captured using a Nikon E200 light microscope (Nikon, Tokyo, Japan).

### 2.10. Docking Simulation Analysis

The three-dimensional structure of LmSCP-2 was predicted using AlphaFold 3 [40]. Model confidence was evaluated using the pLDDT score and predicted TM (pTM) score. Cholesterol structure was obtained from the PubChem database. Model quality was assessed using MolProbity (http://molprobity.biochem.duke.edu/, accessed on 9 April 2026) [41] and Verify3D (https://www.doe-mbi.ucla.edu/verify3d/, accessed on 9 April 2026). Structural homologs were identified using the DaliLite v.3 program [42]. Molecular docking was performed using Discovery Studio 2021 (BIOVIA) and visualized with PyMOL (v4.6) to identify potential cholesterol-binding sites.

### 2.11. Bio-Layer Interferometry Assay

Affinity experiments for LmSCP-2 and cholesterol (Beyotime Biotechnology, Shanghai, China; ST1155) were performed using the Bio-layer interferometry (BLI) system (Gator Bio, Palo Alto, CA, USA). Protein (3 mg/mL) was immobilized on Anti-His Probes with BLI Buffer (0.02%(*v*/*v*) Tween 20 in PBS) at 30 °C. Following a baseline equilibration period of 120 s, probes were incubated with different concentrations of cholesterol for 200 s to monitor association, followed by a 300 s dissociation in BLI Buffer. The results of this assay were analyzed using the GatorOne Data Analysis Module (GatorOne Software v2.18, Gator Bio) by global fitting with a 1:1 binding model. Primers used for generating LmSCP-2 mutant proteins are listed in Table S5.

### 2.12. Statistical Analysis

All experiments were performed with three independent biological replicates. Data are presented as mean ± standard deviation (SD). Statistical comparisons between two groups were performed using a Student’s *t*-test (* *p* < 0.05, ** *p* < 0.01, ns, not significant). For multiple group comparisons, one-way ANOVA followed by Tukey’s multiple comparisons test was used. Different letters indicate statistically significant differences (*p* < 0.05), whereas identical letters indicate no significant difference (ns, *p* > 0.05). Analyses were conducted using GraphPad Prism v9.5.0. Exact *p* values for all comparisons are provided in the figure legend.

## 3. Results

### 3.1. The Expression of LmSCP-x in the Midgut and Fat Body of Locusts

**Figure 1:**
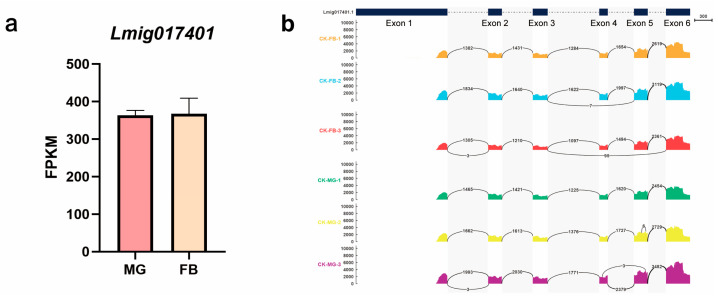
Identification and genomic structure of LmSCP-x in *Locusta migratoria*. (**a**) Expression of *LmSCP-x* in the RNA-seq integument transcriptome dataset of *L. migratoria* (*Lmig017401*). (**b**) Genomic structure of *LmSCP-x*, showing exon–intron organization and alternative splicing patterns across different samples, as well as the predicted transcript variants.

The *LmSCP-x* gene (*Lmig017401*) was identified from transcriptome datasets of the locust midgut and fat body and selected for further analysis (Figure 1a). Transcriptomic analysis revealed that *LmSCP-x* was highly expressed in both the midgut and fat body.

Genome analysis showed that *LmSCP-x* spans 3627 bp and contains six exons and five introns, displaying a conserved gene structure (Figure 1b). Based on these findings, subsequent experiments focused on characterizing the molecular features of *LmSCP-x* and investigating its potential role in cholesterol absorption and transport in *L. migratoria*.

### 3.2. Bioinformatic Analysis of LmSCP-x

**Figure 2:**
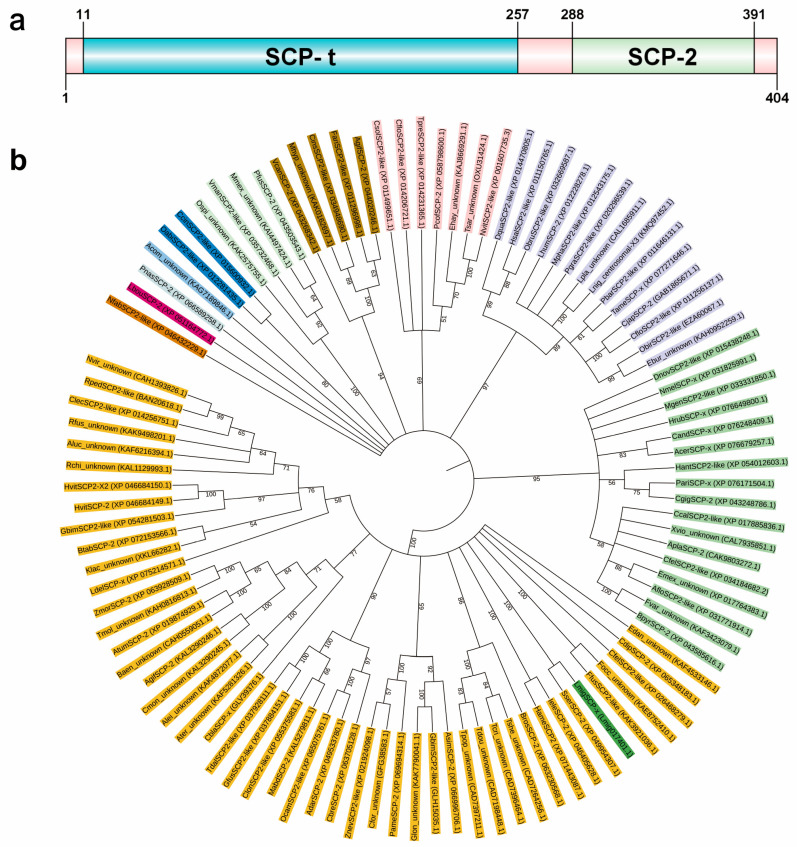
Bioinformatic characterization of LmSCP-x. (**a**) Schematic representation of the predicted protein structure of LmSCP-x. The blue and cyan boxes indicate the SCP-t domain and the SCP-2 domain, respectively. (**b**) Phylogenetic tree of SCP-x proteins from representative insect species constructed using the neighbor-joining method. Different colors represent different insect species or groups. Bootstrap values (%) are indicated at the nodes.

The full-length cDNA sequence of *LmSCP-x* was confirmed using transcriptome data from *L. migratoria*. The gene contains a 1212 bp open reading frame (ORF) encoding a 404-amino-acid protein with a predicted molecular weight of 43.81 kDa and an isoelectric point (pI) of 7.14. Conserved domain analysis revealed that the LmSCP-x protein contains two characteristic domains: a 3-oxoacyl-CoA thiolase (SCP-t) domain and a sterol carrier protein 2 (SCP-2) domain (Figure 2a, Supplementary Figure S1).

Phylogenetic analysis of SCP-x proteins from multiple insect species showed that LmSCP-x (Lmig017401.1) clustered closely with the SCP-x protein of *Schistocerca serialis cubense* (XP049954307.1), indicating a high degree of evolutionary conservation (Figure 2b).

### 3.3. Expression Pattern and Tissue Localization of LmSCP-x

**Figure 3:**
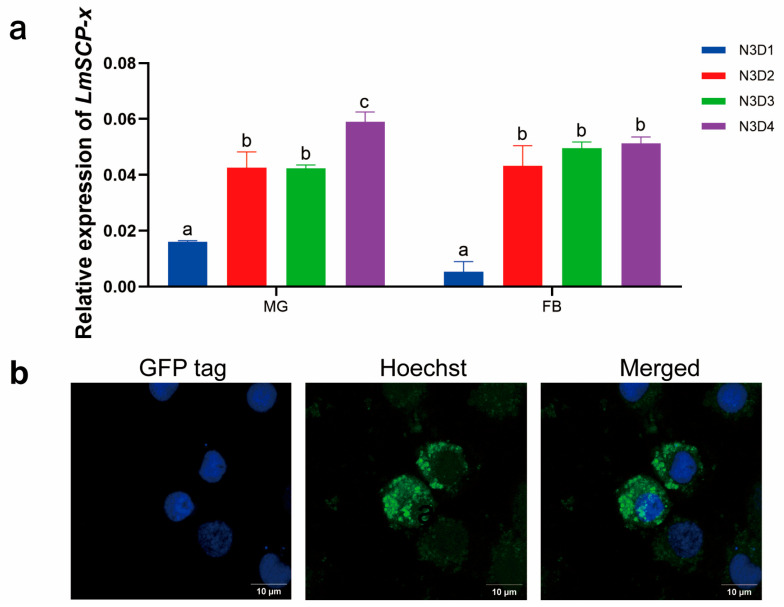
Expression patterns and subcellular localization of LmSCP-x. (**a**) Tissue-specific and integument developmental stage expression profiles of LmSCP-x. FB, fat body; MG, midgut. N3 represents the third-instar nymphs, while D1–D4 represent the first to fourth day. Data are presented as mean ± SD of three biological replicates. Different lowercase letters above the bars indicate significant differences among tissues or developmental stages. In MG: N3D1 vs. N3D2, *p* < 0.05; N3D1 vs. N3D3, *p* < 0.05; N3D1 vs. N3D4, *p* < 0.05; N3D2 vs. N3D3, *p* = 0.9989 (ns); N3D2 vs. N3D4, *p* < 0.05; N3D3 vs. N3D4, *p* < 0.05. In FB: N3D1 vs. N3D2, *p* < 0.05; N3D1 vs. N3D3, *p* < 0.05; N3D1 vs. N3D4, *p* < 0.05; N3D2 vs. N3D3, *p* = 0.2267 (ns); N3D2 vs. N3D4, *p* = 0.0917 (ns); N3D3 vs. N3D4, *p* = 0.9390 (ns). (**b**) Subcellular localization of LmSCP-x-GFP fusion protein expressed in Hi5 cells. GFP fluorescence was observed using confocal microscopy. Cell nuclei were stained with Hoechst 33342 (1 μg/mL). Data are presented as mean ± SD of three independent biological replicates. Statistical significance was determined using one-way ANOVA followed by Tukey’s multiple comparisons test. Different letters indicate statistically significant differences (*p* < 0.05), whereas identical letters indicate no significant difference (ns).

The tissue-specific expression pattern of *LmSCP-x* was examined in ten locust tissues using RT-qPCR. The results showed that *LmSCP-x* was predominantly expressed in the midgut and fat body (Figure 3a). We further analyzed the expression dynamics of *LmSCP-x* during integument development. RT-qPCR results indicated that *LmSCP-x* expression reached its highest level at the late stage of the third-instar nymph, whereas the lowest expression was observed at the early stage (Figure 3a).

To determine the subcellular localization of LmSCP-x, the LmSCP-x open reading frame was fused with GFP, and the fusion protein was expressed in Hi5 cells. Fluorescence microscopy showed that LmSCP-x–GFP signals were mainly distributed in the cytoplasm (Figure 3b). To further verify this localization in insect tissues, the LmSCP-2 domain was expressed and purified (Supplementary Figure S2a,b) and used to generate specific antibodies. Immunohistochemical analysis of locust tissues confirmed that SCP-x protein was primarily localized in the cytoplasm, consistent with the in vitro cell localization results (Supplementary Figure S2c).

### 3.4. LmSCP-x Is Involved in Cholesterol Absorption and Transport in Locusts

**Figure 4:**
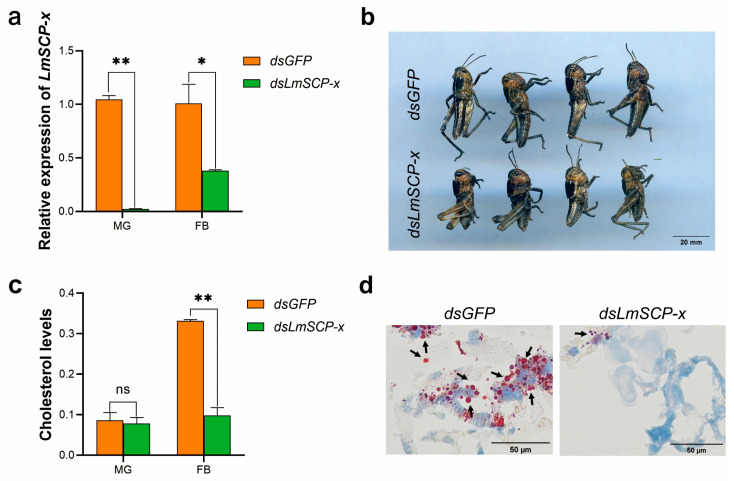
Functional analysis of LmSCP-x by RNA interference. (**a**) Silencing efficiency of LmSCP-x in the midgut (MG) and fat body (FB) following dsRNA injection. Exact *p* values for each comparison are indicated: MG, *dsGFP* vs. *dsLmSCP-x*, *p* < 0.01; FB, *dsGFP* vs. *dsLmSCP-x*, *p* = 0.0283. (**b**) Growth and developmental phenotypes of *L. migratoria* nymphs after dsRNA injection. (**c**) Cholesterol levels in the MG and FB after treatment with dsGFP or dsLmSCP-x. Exact *p* values for each comparison are indicated: MG, *dsGFP* vs. *dsLmSCP-x*, *p* = 0.5979; FB, *dsGFP* vs. *dsLmSCP-x*, *p* < 0.01. (**d**) Effect of LmSCP-x knockdown on lipid accumulation in the fat body of *L. migratoria* nymphs as detected by Oil Red O staining. Black arrows indicate lipid droplets stained by Oil Red O. Data are presented as mean ± SD of three independent biological replicates. Statistical significance was determined using Student’s *t*-test (* *p* < 0.05, ** *p* < 0.01, ns, not significant).

The efficiency of RNAi-mediated *LmSCP-x* knockdown was evaluated by RT-qPCR at 48 h after dsRNA injection. Treatment with dsLmSCP-x significantly reduced *LmSCP-x* transcript levels (Figure 4a), with reductions of 93.01% and 70.03% compared with the control group. These results confirmed that RNAi was effective for the functional analysis of *LmSCP-x*.

To investigate the role of LmSCP-x in cholesterol metabolism, RNAi-mediated gene silencing was combined with cholesterol quantification assays. As shown in Figure 4b, locusts injected with dsLmSCP-x exhibited slower growth and development, and the developmental delay rate increased by 70.86% compared with the dsGFP control group.

Given the known role of SCP-x proteins in sterol transport, we hypothesized that LmSCP-x influences locust development by regulating sterol transport and storage. To test this hypothesis, cholesterol levels in the midgut and fat body were measured following RNAi treatment.

As shown in Figure 4c, knockdown of *LmSCP-x* resulted in a ~60% reduction in cholesterol content in the fat body compared with the *dsGFP* control (*p* < 0.01). These results indicate that LmSCP-x plays an important role in cholesterol absorption and transport in locusts. To further examine whether LmSCP-x affects lipid accumulation in the fat body, Oil Red O staining was performed on frozen sections to visualize lipid deposition. As shown in Figure 4d, the intensity of red staining on the surface of nymphal sections from *dsLmSCP-x*-treated locusts was noticeably weaker than that observed in the *dsGFP* control group. Quantitative analysis of the staining intensity is shown in Supplementary Figure S2d, confirming a significant reduction in lipid accumulation upon *LmSCP-x* knockdown. These results suggest that silencing *LmSCP-x* reduces lipid accumulation in the fat body.

### 3.5. Ligand-Binding Properties of LmSCP-2 in L. migratoria

**Figure 5:**
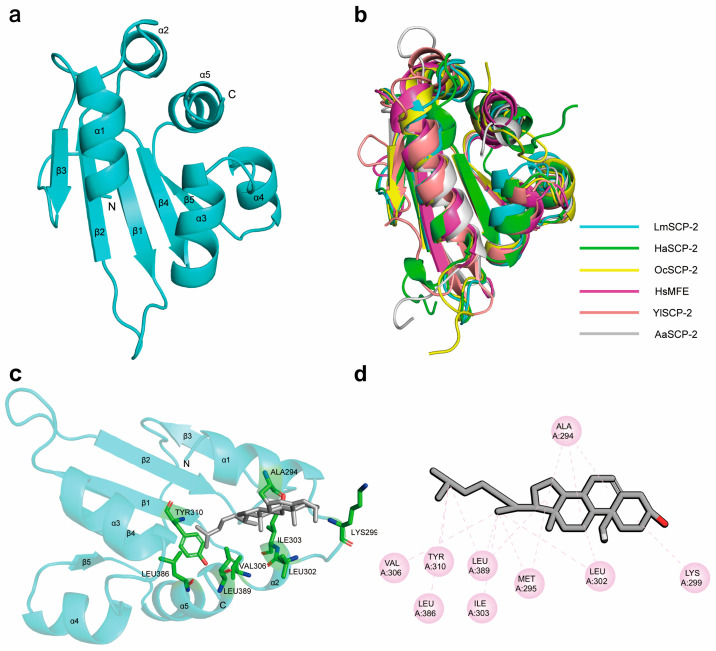
Structural analysis of the SCP-2 domain and its interaction with sterol. (**a**) Predicted three-dimensional model of LmSCP-2, with the protein backbone shown as a blue cartoon. (**b**) Structural superposition of LmSCP-2 with SCP-2 proteins from other species, where different colored cartoons represent proteins from different species. (**c**) Overall view of the sterol-binding site. Sterol is shown in gray as a stick model, and the protein backbone is represented as a blue cartoon. (**d**) Close-up three-dimensional view of the sterol-binding pocket. Key surrounding amino acid residues are labeled in black, and pink dashed lines indicate hydrophobic interactions.

The three-dimensional structure of LmSCP-2 was predicted using AlphaFold 3, revealing a hydrophobic ligand-binding cavity (Figure 5a). The predicted template modeling (pTM) score was 0.81, suggesting a high-confidence and reliable global structural model (Supplementary Figure S3). Similar to SCP-2 proteins from cotton bollworm, rabbit, human, and mosquito, LmSCP-2 exhibits a typical α/β fold arrangement (Figure 5b), which is a conserved structural feature of the sterol carrier protein family. The secondary structure elements are arranged in the order α1–α2–β1–β2–β3–β4–α3–α4–β5–α5. The protein core consists of a four-stranded β-sheet (strand order 3–2–4–1) covered by five α-helices, forming a large hydrophobic cavity capable of accommodating sterol or lipid molecules.

Structural similarity analysis using the DALI program identified several homologous proteins belonging to the SCP-2 family with sequence identities ranging from 19% to 77% and root-mean-square deviation (RMSD) values of 1.4–2.3 Å (Table 1). The most similar structure corresponded to *Helicoverpa armigera* SCP-2 (PDB: 4uei) with 77% sequence identity and an RMSD of 1.9 Å. The *Oryctolagus cuniculus* SCP-2 (PDB: 1c44) also showed structural similarity with 52% sequence identity and an RMSD of 1.4 Å. These results were consistent with the sequence alignment analysis shown in Figure S7. A structural difference between LmSCP-2 and AeSCP-2 was observed in the absence of a loop between α-helix 1 and β-sheet I, which affects the opening of the ligand-binding cavity (Figure 5b).

The Ramachandran plot indicated that 98.0% of residues were located in favored regions, and 100% were within allowed regions, with no outliers (Supplementary Figure S4). In addition, 85.58% of residues exhibited 3D–1D scores greater than 0.1 in Verify3D analysis (Supplementary Figure S5), supporting the reliability of the predicted model. Pocket analysis identified multiple hydrophobic residues lining the binding cavity, including MET291, ALA294, MET295, ASP298, LEU302, ILE303, VAL306, ARG307, GLY308, TYR310, PHE312, ILE327, ASN328, ALA329, LYS333, GLY334, LEU382, LYS385, LEU386, and LEU389, which may participate in sterol or lipid binding (Supplementary Figure S8). Molecular docking analysis using the LibDock program in Discovery Studio 2021 revealed a predicted LibDockScore of 100 for cholesterol binding. Cholesterol formed hydrophobic interactions with residues ALA294, MET295, LEU302, ILE303, VAL306, TYR310, LEU386, and LEU389 in the binding cavity (Figure 5c,d), suggesting that LmSCP-2 may directly interact with cholesterol molecules. Biolayer interferometry (BLI) assays confirmed this interaction, with a measured binding affinity (*K_D_*) of 1.31 ± 1.02 µM. Mutation of LEU389 to alanine (L389A) abolished cholesterol binding, indicating that LEU389 is critical for the interaction (Supplementary Figure S9).

## 4. Discussion

Sterol carrier protein x/2 (SCP-x/2) represents an evolutionarily conserved class of nonspecific lipid transport proteins that participate in intracellular cholesterol transport, metabolic regulation, and lipid homeostasis. Despite extensive studies in vertebrates and several insect taxa, the biological role of SCP-x in Orthopteran insects remains largely unexplored. In the present study, transcriptomic analysis of the migratory locust (*L*. *migratoria*) identified an *SCP-x* gene, and its potential role in lipid transport was investigated. Phylogenetic analysis showed that *LmSCP-x* clusters closely with *SCP-x* from *S*. *serialis cubens*, suggesting evolutionary conservation of SCP-x proteins within Orthoptera. Consistent with this observation, expression analysis demonstrated that LmSCP-x is predominantly expressed in the midgut and fat body, two key tissues involved in nutrient absorption and lipid metabolism in insects.

Functional analysis further revealed that RNAi-mediated silencing of *LmSCP-x* significantly affected locust development and cholesterol metabolism. Knockdown of *LmSCP-x* increased the diapause rate of locusts to approximately 70% and reduced cholesterol levels in the fat body by 30%, indicating that LmSCP-x plays an important role in cholesterol transport in *L. migratoria*. Similar functional roles of SCP-2 proteins have been reported in other organisms. For example, overexpression of the 13 kDa SCP-2 in mouse hepatocytes increased intracellular cholesterol levels by approximately 70%, while reducing endogenous cholesterol synthesis by about 60% [43]. Conversely, gene knockout studies in mice showed that total hepatic cholesterol levels decreased by approximately 15%, mainly due to a reduction in cholesteryl esters [44]. In insects, RNA interference-mediated knockdown of SCP-2 expression in mosquitoes significantly reduced cellular cholesterol uptake [45,46]. Similar results were also reported in the *Spodoptera litura* Spli-221 cell line, where RNAi-mediated suppression of SCP-2 decreased cholesterol uptake, whereas SCP-2 overexpression markedly enhanced intracellular cholesterol accumulation [14]. These findings collectively support the conserved role of SCP-2 family proteins in regulating intracellular sterol transport.

Structural analysis further demonstrated that LmSCP-x possesses the characteristic features of insect SCP-x proteins, including the SCP-2 domain. In this study, the three-dimensional structure of LmSCP-2 from the orthopteran insect *L. migratoria* was predicted using AlphaFold 3. Structural comparison with other SCP-2 proteins, including *Homo sapiens* SCP-2 (1ikt), *Oryctolagus cuniculus* SCP-2 (1c44), *Aedes Aegypti* SCP-2 (1pz4), *Helicoverpa armigera* SCP-2 (4uei), and *Yarrowia lipolytica* SCP-2 (4jgx), revealed that members of the SCP-2 family share a highly conserved α/β-fold architecture composed of four to five α-helices and four to five β-strands. This conserved structural organization suggests that SCP-2 proteins maintain similar ligand-binding mechanisms across both vertebrates and invertebrates. The structural framework formed by α-helices and β-strands is likely essential for the ligand-binding activity of SCP-2 proteins.

Further structural analysis indicated that LmSCP-2 contains a hydrophobic binding cavity capable of accommodating small hydrophobic molecules such as sterols and lipids. Notably, LmSCP-2 exhibited the highest structural similarity to the SCP-2 protein of the lepidopteran insect *H*. *armigera*, whereas its similarity to mosquito SCP-2 proteins was comparatively lower (Figure 5; Table 1 and Supplementary Figure S6). Similar to *H. armigera* SCP-2, *L. migratoria* SCP-2 contains five α-helices, while mosquito AeSCP-2 contains only four α-helices and lacks the α-2 helix that is present in both *H. armigera* SCP-2 and *L. migratoria* SCP-2. In contrast to *H. armigera* SCP-2, however, the *L. migratoria* SCP-2 structure includes a supporting network composed of five β-strands, whereas *H. armigera* SCP-2 contains only four β-strands. These structural differences among species may influence ligand recognition and binding properties. Importantly, such variations may suggest potential opportunities for the development of species-specific chemical inhibitors targeting SCP-2 proteins; however, this interpretation remains speculative and requires further experimental validation.

Biochemical analyses further showed that LmSCP-2 exists as a monomeric protein in solution, as indicated by gel filtration and sedimentation experiments. This observation is consistent with previous studies reporting that SCP-2 proteins from humans (MsMFE-2), rabbits (OcSCP-2), mosquitoes (AeSCP-2 and AeSCP-2L3), and *H. armigera* also function as monomeric proteins [18,47,48]. These findings suggest that LmSCP-2 likely performs its biological functions in a monomeric form within cells.

Based on the predicted structure of LmSCP-2, the interaction between LmSCP-2 and the ligand cholesterol was further investigated. Molecular docking analysis indicated that cholesterol interacts with several hydrophobic amino acid residues located within the binding cavity of LmSCP-2. These residues include ALA294, MET295, LEU302, ILE303, VAL306, TYR310, LEU386, and LEU389, which correspond to ALA35, MET36, LEU43, ILE44, VAL47, TYR51, LEU127, and LEU130 in HaSCP-2 (Supplementary Figure S7). Previous studies have shown that the Y51A mutation in HaSCP-2 significantly reduces its binding affinity to NBD-cholesterol, indicating that these residues play critical roles in hydrophobic interactions between SCP-2 proteins and cholesterol molecules [18].

Taken together, these results suggest that mutations in key residues within the hydrophobic cavity may alter the ligand-binding properties of LmSCP-2, thereby affecting its ability to bind and transport sterol or lipid molecules. In this study, BLI assays confirmed that mutation of LEU389 abolished cholesterol binding, demonstrating the functional importance of this residue. From an applied perspective, such critical residues may serve as potential molecular targets for the development of novel insecticides aimed at disrupting cholesterol transport in locusts. Future studies combining additional mutagenesis and biochemical binding assays will be necessary to further validate the roles of other residues and to clarify the molecular mechanisms underlying SCP-2-mediated sterol transport in Orthopteran insects.

## 5. Conclusions

In this study, a sterol carrier protein gene, *LmSCP-x*, was identified and characterized in the migratory locust (*L*. *migratoria*). Expression analysis revealed that *LmSCP-x* is predominantly expressed in the midgut and fat body, suggesting its involvement in lipid metabolism. Functional analysis demonstrated that RNAi-mediated silencing of *LmSCP-x* significantly reduced cholesterol levels in the fat body and increased the developmental delay rate of locusts, indicating that *LmSCP-x* plays an important role in cholesterol transport and developmental regulation in this species.

Structural prediction further showed that LmSCP-2 contains a conserved α/β-fold architecture and a hydrophobic binding cavity capable of accommodating cholesterol molecules. Molecular docking analysis identified several key residues that may contribute to cholesterol binding. Together, these findings provide new insights into the molecular mechanism of sterol transport in Orthopteran insects. The identified functional residues of LmSCP-2 may represent potential molecular targets for the development of novel pest management strategies against locusts. Future studies focusing on functional mutagenesis and ligand-binding assays will help to further elucidate the biochemical mechanisms underlying SCP-x mediated sterol transport.

## Figures and Tables

**Table 1:** Proteins with similar structures to LmSCP-2.

Organism	PDB	RMSD ^a^	Identities	Method	Resolution	Description
*Helicoverpa armigera*	4uei	1.9 Å	77%	NMR	-	SCP-2
*Oryctolagus cuniculus*	1c44	1.4 Å	52%	X-ray	1.80 Å	SCP-2
*Homo sapiens*	1ikt	2.3 Å	34%	X-ray	1.75 Å	MFE-2
*Yarrowia lipolytica*	4jgx	2.3 Å	27%	X-ray	2.20 Å	SCP-2
*Aedes Aegypti*	1pz4	2.2 Å	19%	X-ray	1.35 Å	SCP-2

^a^ RMSD (The root-mean-square deviation) is the measure of the average distance between the backbone Cα atoms of superimposed proteins.

## Data Availability

All data from the study are available in the Supporting Material or upon demand.

## Supplementary Materials

The following supporting information can be downloaded at: https://www.mdpi.com/article/10.3390/biology15080613/s1, Figure S1: Conserved domains of LmSCP-x; Figure S2: a: The elution profile of LmSCP-2 on a Superdex 75 increase column. b: SDS-PAGE gel of purified LmSCP-2 proteins. c: Localization of LmSCP-x in the MG by immunohistochemistry. Nucleus and LmSCP-x were detected by a green, and red signal, respectively. d: Quantification of Oil Red O staining in FB sections corresponding to Figure 4d; Figure S3: The 3D model of LmSCP-2 was predicted using AlphaFold 3; Figure S4: MolProbity Ramachandran analysis of the constructed 3D model of LmSCP-2; Figure S5: Verify 3D analysis of the constructed 3D model of LmSCP-2; Figure S6: A phylogenetic tree of SCP-2 in insects was constructed with the neighbor-joining method; Figure S7: Alignment of SCP-2 domain sequences from several species; Figure S8: Schematic showing the hydrophobic residues on the pocket of LmSCP-2 domain, residues surrounding the cavity are highlighted with green color; Figure S9. BLI analysis of LmSCP-2 binding to cholesterol. Table S1: Sequence of primers used for expression of LmSCP-x-GFP in Hi5 cells; Table S2: Sequence of primers used for expression of LmSCP-2 fragments in *E. coli* DE3; Table S3: Double-stranded RNA primers; Table S4: Sequences of the primers used for real time PCR analyzes. Table S5: Sequences of the primers used for site-directed mutagenesis of LmSCP-2.
