# Supplementary material for: Sterol Carrier Protein X (SCP-x) Regulates Cholesterol Transport in the Migratory Locust Locusta migratoria
Source: Biology (Basel). 2026 Apr 13;15(8):613. doi: 10.3390/biology15080613 (PMC13113942; doi:10.3390/biology15080613)
Supplement: Supplementary file 1 [file biology-15-00613-s001.zip › biology-4233873-supplementary.pdf]

Supplementary information：

Sterol Carrier Protein X (SCP-x) Regulates Cholesterol Transport in the Migratory Locust *Locusta migratoria*

Dengbo Li [1], Tian Miao [1], Zige Wang [1], Zimeng Lang [1], Zixin Wang [1], Zixuan Zhou [1], Jinming Zhao [1], Panting Ma [1] and Yuemin Ma [1,2,*]

[1] College of Life Science, Shanxi University, Taiyuan 030006, China
[2] School of Synthetic Biology, Shanxi University, Taiyuan 030006, China
* Correspondence: ymma@sxu.edu.cn; Tel.: +86-130-0710-2085

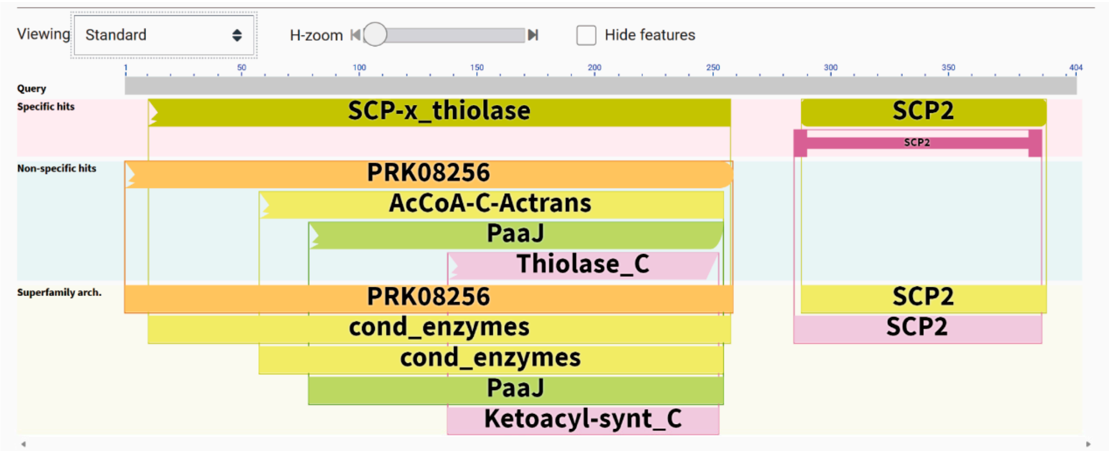

Name	Accession	Description	Interval	E-value
PRK08256	PRK08256	lipid-transfer protein	1-258	1.08×10^{-167}
SCP-x_thiolase	cd00829	Thiolase domain associated with sterol carrier protein (SCP)-x isoform and related proteins	11-257	3.14×10^{-113}
SCP2	pfam02036	SCP-2 sterol transfer family	288-391	2.13×10^{-25}
SCP2	COG3255	Putative sterol carrier protein, contains SCP2 domain [Lipid transport and metabolism]	285-389	2.21×10^{-19}
AcCoA-C-Actrans	TIGR01930	acetyl-CoA acetyltransferases	58-254	7.23×10^{-15}
PaaJ	COG0183	Acetyl-CoA acetyltransferase [Lipid transport and metabolism]	79-254	1.38×10^{-14}
Thiolase_C	pfam02803	Thiolase, C-terminal domain	138-252	1.00×10^{-8}

Figure S1. Conserved domains of *LmSCP-x*.

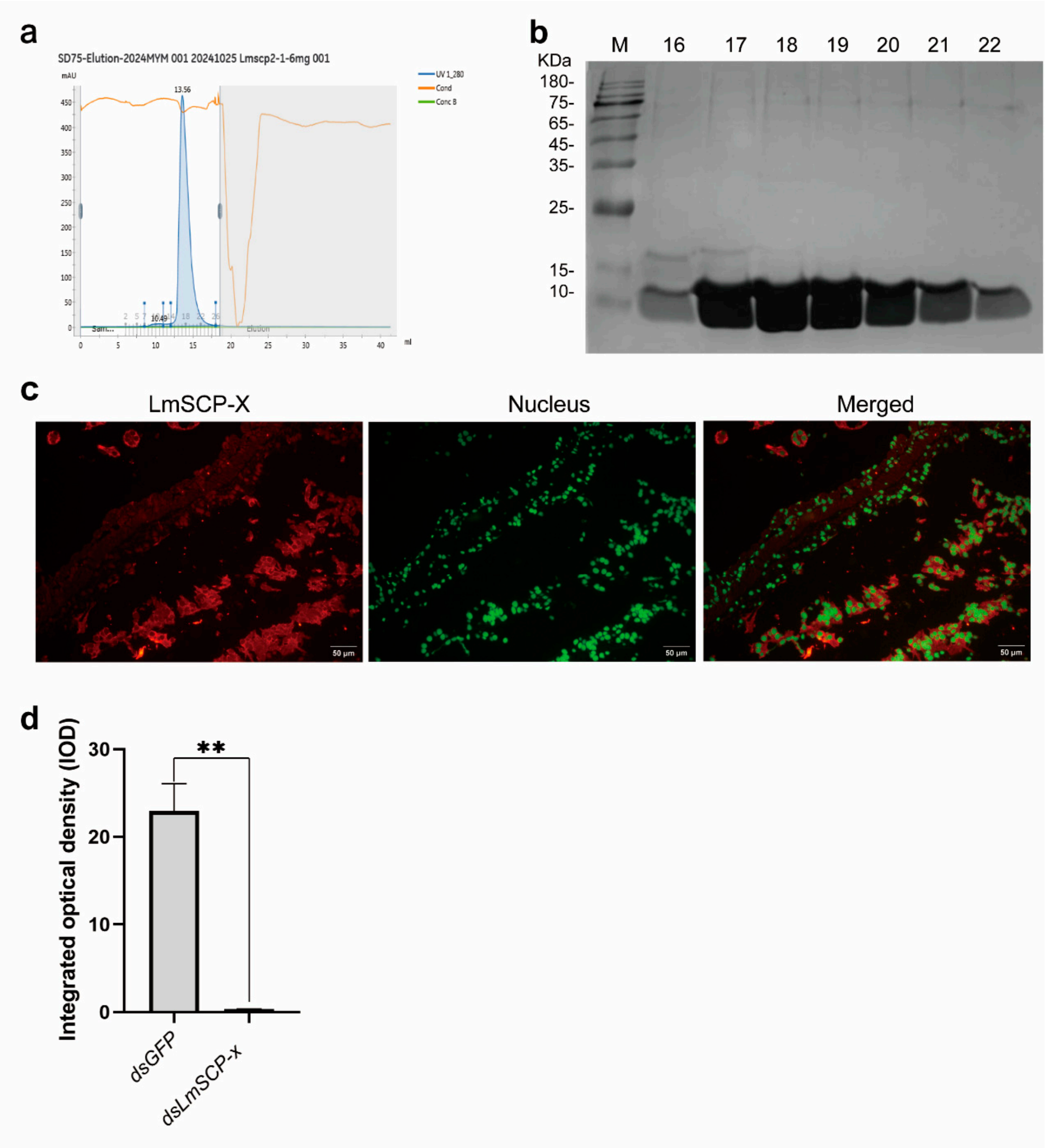

Figure S2. a: The elution profile of LmSCP-2 on a Superdex 75 increase column. b: SDS-PAGE gel of purified LmSCP-2 proteins. c: Localization of LmSCP-x in the MG by immunohistochemistry. Nucleus and LmSCP-x were detected by a green, and red signal, respectively. d: Quantification of Oil Red O staining in FB sections corresponding to Figure 4d. Data are presented as mean ± SD of three independent biological replicates. Statistical significance was determined using Student's t-test (** $p < 0.01$).

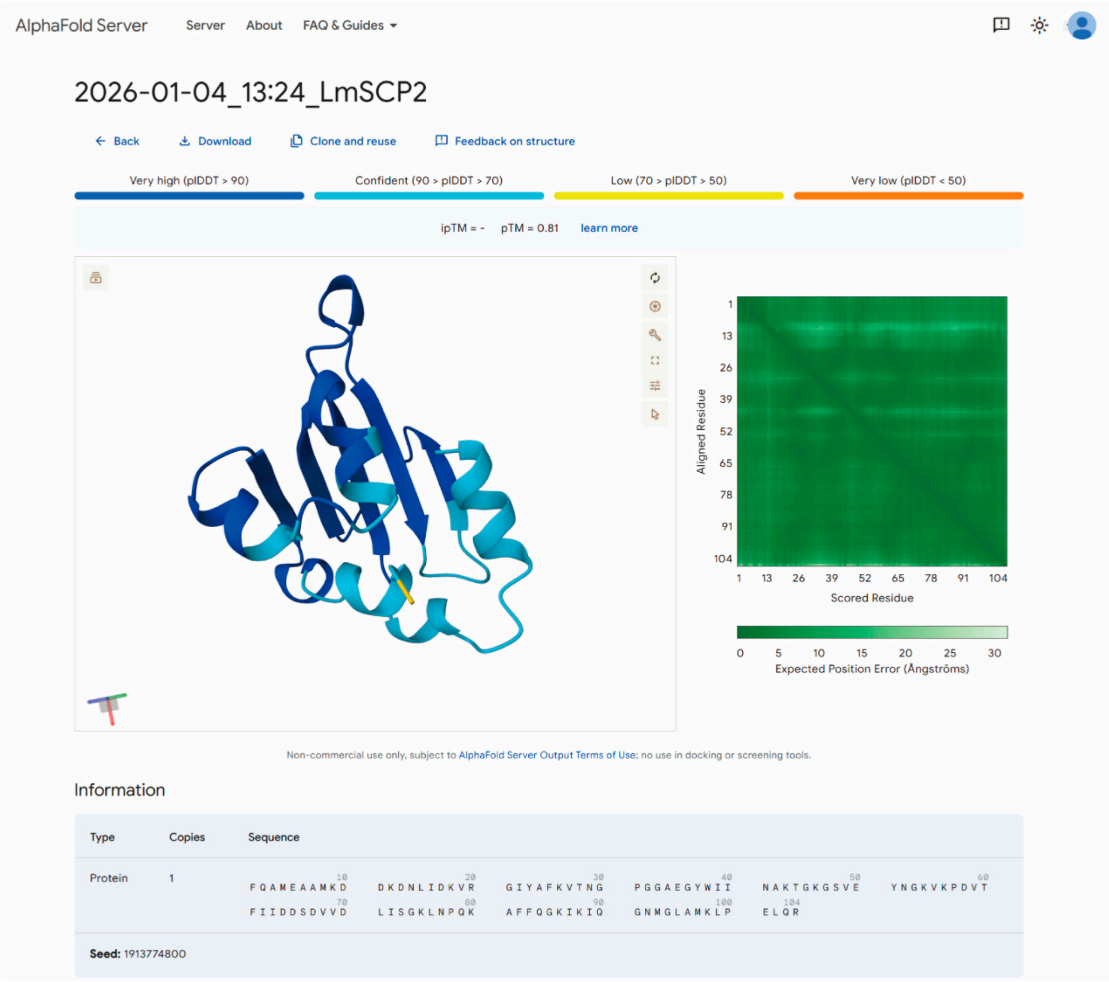

Figure S3. 3D Structural Prediction of LmSCP-2. The 3D model of LmSCP-2 was predicted using AlphaFold 3 to investigate its conserved SCP-2 domain architecture and to identify potential cholesterol-binding sites.

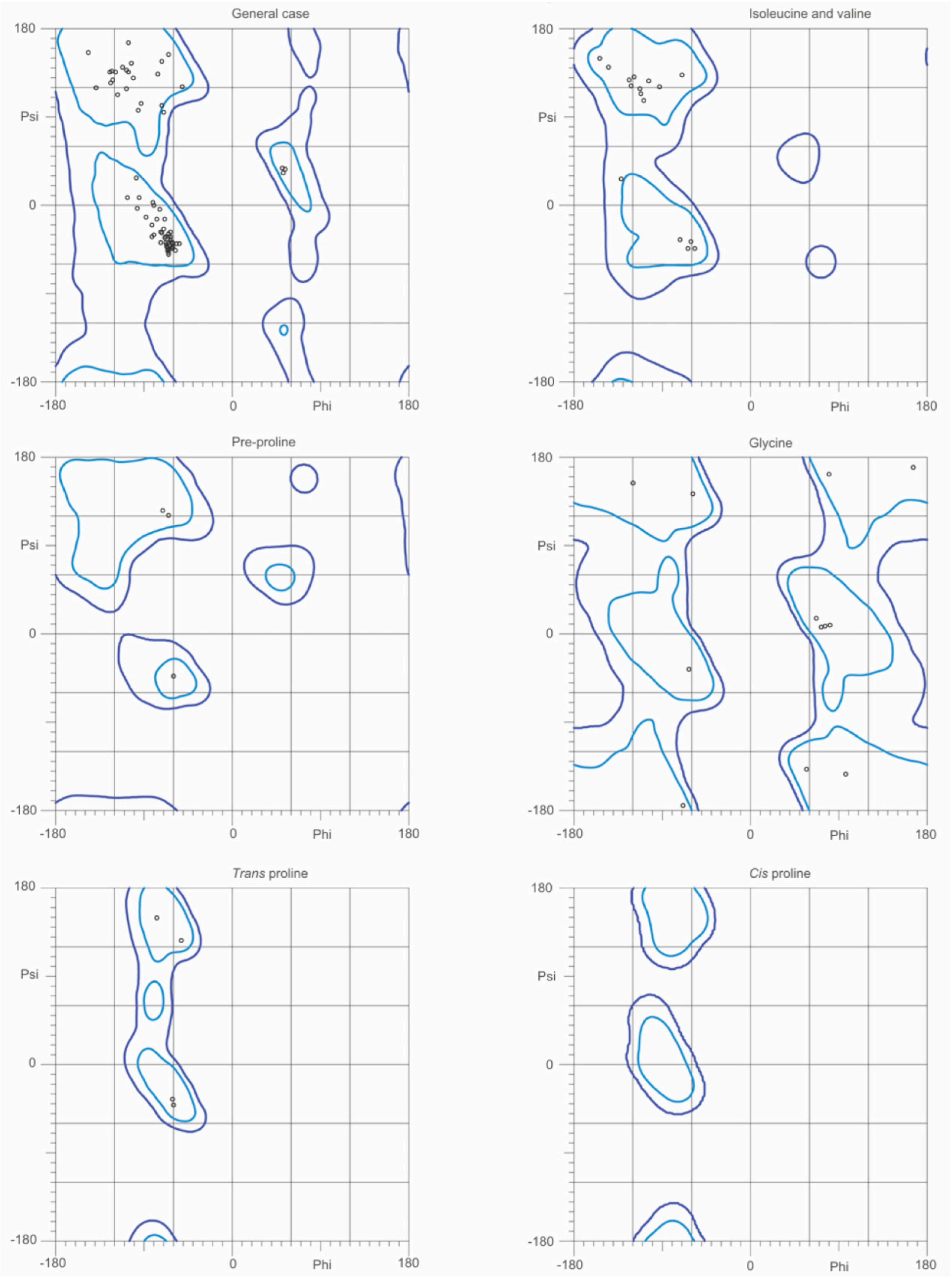

Figure S4. Validation of LmSCP-2 3D Structure via MolProbity Ramachandran Analysis. The constructed 3D model of LmSCP-2 was evaluated using MolProbity Ramachandran analysis to assess the stereochemical quality and reliability of the predicted structure, ensuring that most residues fall within

favored and allowed regions.

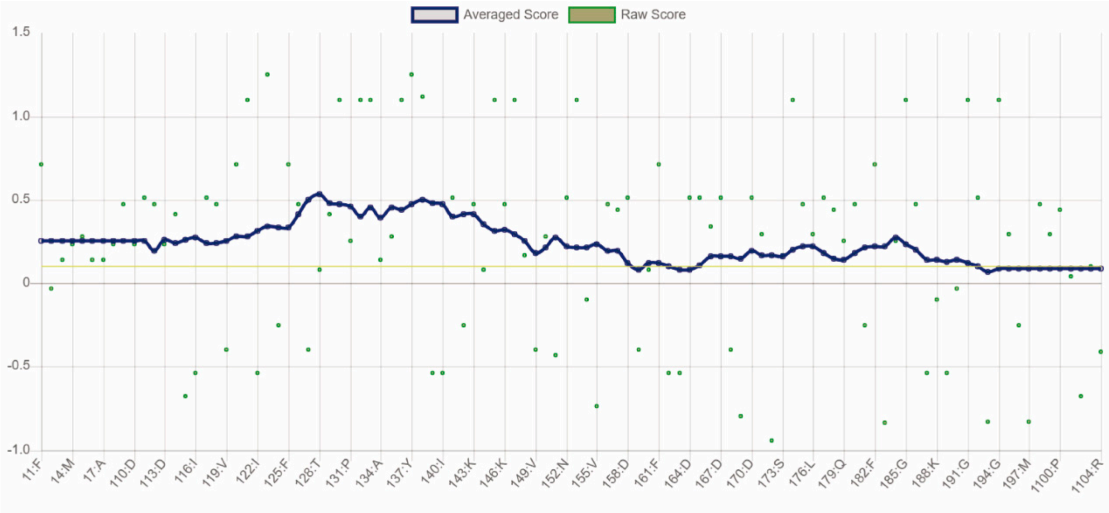

Figure S5. Validation of LmSCP-2 3D Structure via Verify 3D analysis. This complementary assessment confirmed that the overall folding and residue environments were consistent with expected patterns, providing additional confidence in the model's suitability for downstream molecular docking and functional studies.

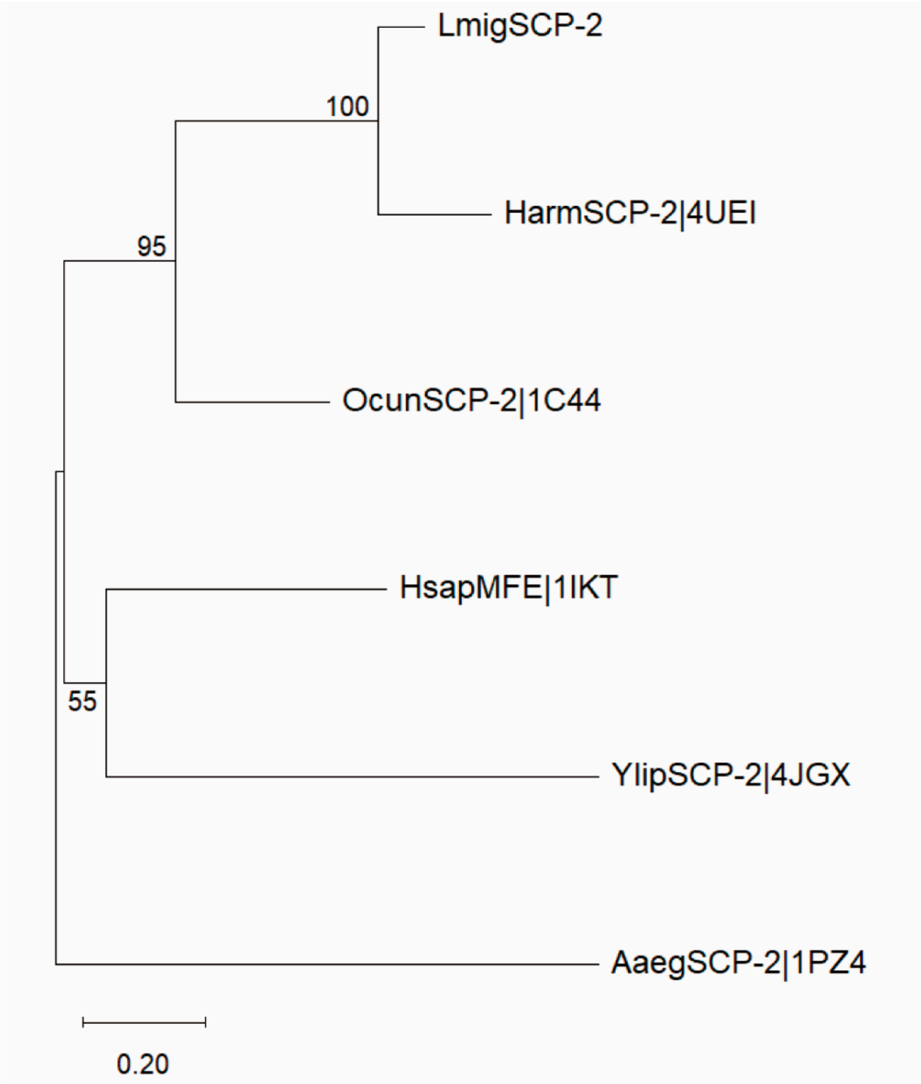

Figure S6. A phylogenetic tree of SCP-2 in insects was constructed with the neighbor-joining method.

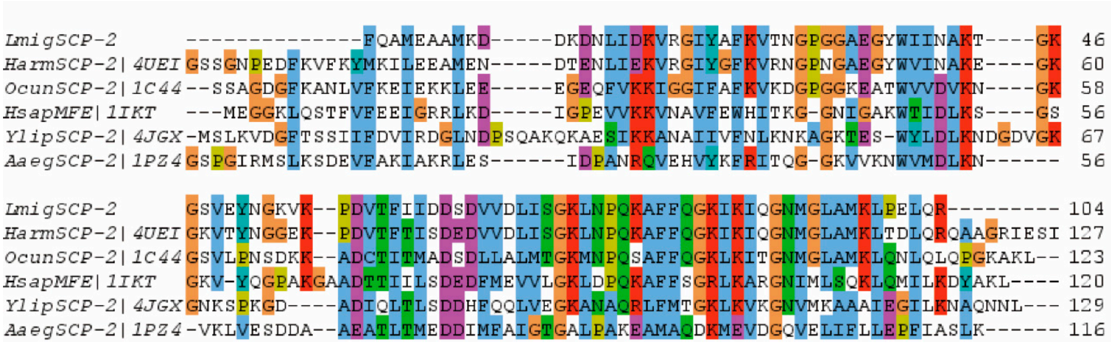

Figure S7. Alignment of SCP-2 domain sequences from several species.

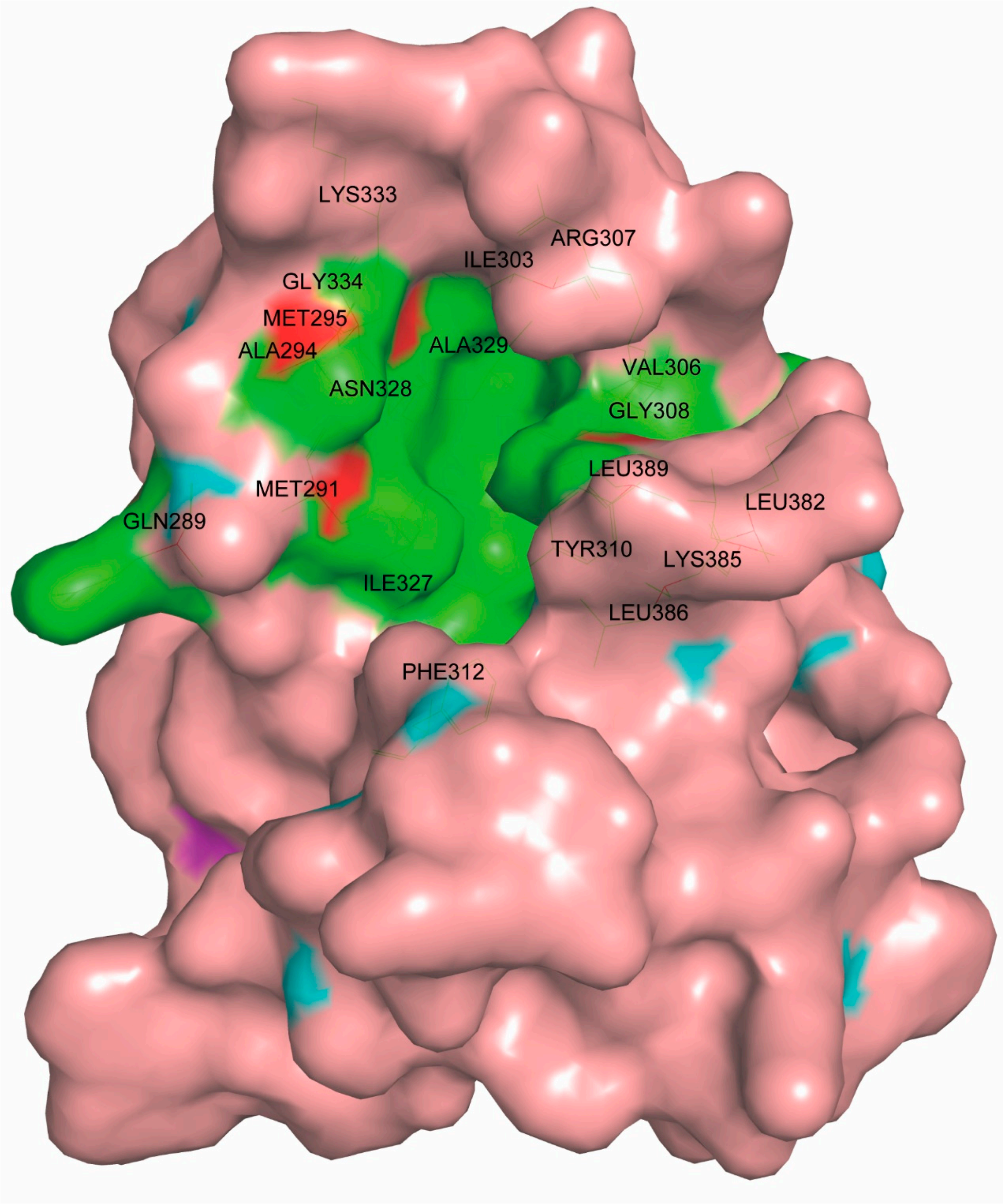

Figure S8. Schematic showing the hydrophobic residues on the pocket of LmSCP-2 domain, residues surrounding the cavity are highlighted with green color.

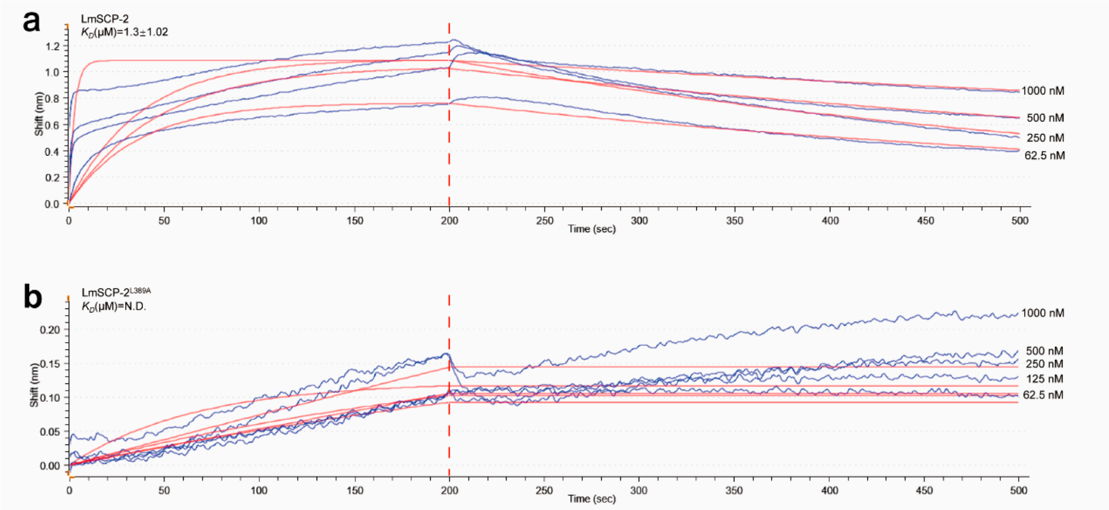

Figure S9. BLI analysis of LmSCP-2 binding to cholesterol. a: Binding curves of wild-type LmSCP-2 to cholesterol at different concentrations. b: Binding curves of the LmSCP-2 mutant L389A with cholesterol. The L389A mutation abolished cholesterol binding, demonstrating the functional importance of LEU389. Blue lines represent experimental data at each cholesterol concentration, and red lines indicate the global fitting. The K_D was calculated from the fitted curves. Data shown are representative of three independent experiments.

Table S1. Sequence of primers used for expression of LmSCP-x-GFP in Hi5 cells

Primer	Primer sequences (5′-3′)
Insert-F	TCGAGCTCAAGCTTCGGCCACCATGGCAGACATTGCAGGCATAA
Insert-R	TGGTGGCGACCGGTGGATCTAACTTTGACTTGAACTCCTCAATT
Vector-F	GATCCACCGGTCGCCACCA
Vector-R	CGAAGCTTGAGCTCGAGATCT

Table S2. Sequence of primers used for expression of LmSCP-2 fragments in *E.coli DE3*

Primer	Primer sequences (5′-3′)
His-F	AGAACAGATTGGTGGATCCTTCCAAGCTATGGAAGCCGCAATGA
His-R	AGCATTATGCGGCCGCTTACCTCTGAAGTTCAGGAAGTTTCATT
KS-F	TAAGCGGCCGCATAATGCTTAAGT
KS-R	GGATCCACCAATCTGTTCTCTGTGAGC

Table S3. Double-stranded RNA primers

Primer	Primer sequences (5′-3′)
dsEF-1α-F	TAATACGACTCACTATAGGGTGGAGAGGGTGAAGG
dsEF-1α-R	TAATACGACTCACTATAGGGGGGCAGATTGTGTGGAC
dsLmSCP-x-F	TAATACGACTCACTATAGGGTATAGGCTTGGTTTCCCAAGGC
dsLmSCP-x-R	TAATACGACTCACTATAGGGTAACTTTGACTTGAACTCCTCA

Table S4. Sequences of the primers used for real time PCR analyzes

Primer	Primer sequences (5′-3′)
EF-1α-F	AGCCCAGGAGATGGGTAAAG
EF-1α-R	CTCTGTGGCCTGGAGCATC
LmSCP-x-F	TTGGATAATTAATGCAAAGACGGG
LmSCP-x-R	CTGTGGATTCAGCTTACCAGAT

Table S5. Sequences of the primers used for site-directed mutagenesis of LmSCP-2

Primer	Primer sequences (5′-3′)
MUT389-F	ATGAAACTTCCTGAAGCTCAGAGGCGA
MUT389-R	GCTTCAGGAAGTTTCATTGCTAGTCC
